# Unusual pediatric co-morbility: autoimmune thyroiditis and cortico-resistant nephrotic syndrome in a 6-month-old Italian patient

**DOI:** 10.1186/1824-7288-38-57

**Published:** 2012-10-23

**Authors:** Flavia Urbano, Angelo Acquafredda, Gabriella Aceto, Rosa Penza, Luciano Cavallo

**Affiliations:** 1 Biomedicine Department of Development Age, University of Bari “Aldo Moro”, Piazza Giulio Cesare, 11, 70124, Bari, Italy

**Keywords:** Autoimmune thyroiditis, Nephrotic syndrome, Childhood

## Abstract

We report on a case of autoimmune thyroiditis in a 6-month-old patient with cortico-resistant nephrotic syndrome. Normal serum levels of thyroid hormons and thyroid-stimulating hormone were detected with high titers of circulant antithyroid antibodies and a dysomogeneous ultrasound appearance of the gland, typical of autoimmune thyroiditis. The research of maternal thyroid antibodies was negative. This is the first case of autoimmune thyroiditis found in such a young patient with pre-existing nephrotic syndrome ever described in literature. This association is random because nephrotic syndrome does not have an autoimmune pathogenesis and the genes involved in autoimmune thyroiditis are not related to those of nephrotic syndrome.

## Introduction

Autoimmune diseases are very rare conditions in infancy, especially in the first year of life [1-4]. We report on a case of unusual early-onset autoimmune thyroiditis in a 6-month-old child affected by nephrotic syndrome. Before that, a case of isolated autoimmune thyroiditis has been reported previously at a so early age: the patient, a 7-month-old child, died at the age of 9 months for a sepsis and his autopsy revealed a thyroid atrophy [1].

## Case report

**Figure 1:**
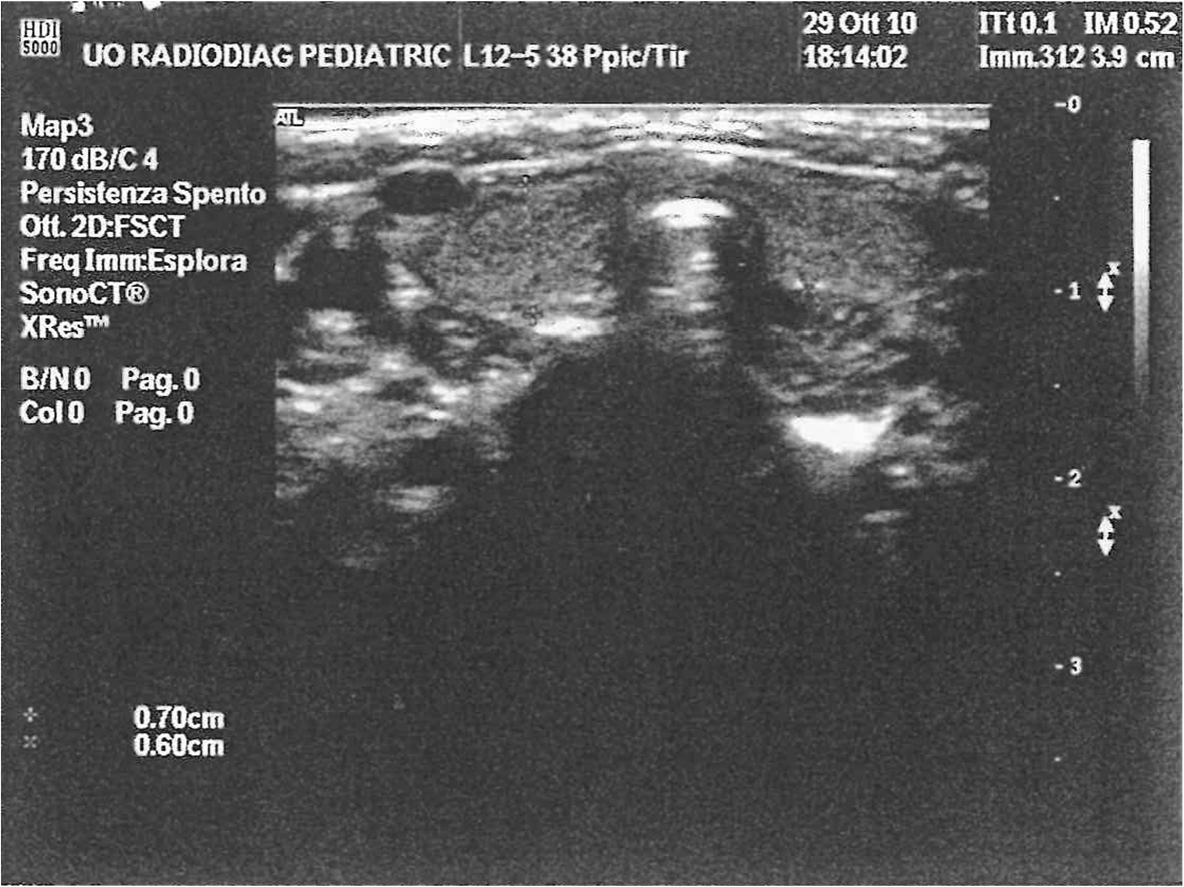
Ultrasound image of thyroiditis in a 6-month-old child.

D.S. was admitted to our hospital at 6 months of age because of the appearance, two weeks before, of bilateral periorbital oedema associated with hypoalbuminemia (albumin 0.5 g/dl), hypoprotidemia (seric proteins 3.7 g/dl), proteinuria (proteinuria/creatinuria ratio 114,3) and dyslipidemia (total cholesterolemia 287 mg/dl, LDL-cholesterol 165 mg/dl, triglycerides 997 mg/dl). The diagnosis of nephrotic syndrome was established and a substitutive therapy including human albumin was immediately started, as well as calcium and potassium supplementation, diuretics and intravenous immunoglobulines. After a few days albumin levels further decreased, general conditions became more serious and subcutaneous succulence spread especially in abdominal, scrotal and palpebral regions. For this reason, it became necessary to intensify albumin infusion as well as diuretic supply and to introduce prednisone (starting dose 1 mg/Kg/die, than 0,5 mg/Kg/die) in association with an ACE-inhibitor (dose 0,5 mg/Kg/die) for its antiproteinuric effect, in order to potentiate steroid action. This therapy was given for overall eight weeks without any response: therefore this form of nephrotic syndrome can be defined “cortico-resistant”. After a few days, for the aggravation of clinical conditions, also cyclophosphamide (dose 1 mg/Kg/die) and spironolactone (potassium-sparing) were introduced unsuccessfully. Blood pressure was always normal. Because of the massive proteinuria, due to the renal failure, an evaluation of thyroid function was made: thyroid hormones were normal but high titers of circulating antithyroglobulin and anti-microsomal thyroid antigen antibodies were detected [5,6]. No antithyroid antibodies were found in his mother, excluding a transplacentar transfer [7]. The diagnosis of autoimmune thyroiditis was confirmed by the typical ultrasound appearance of the gland, characterized by normal morphology and sizes but a dysomogeneous structure (Figure 1).

After a month a blood assay revealed the persistence of high titers of antithyroid antibodies associated to a slight decrease of thyroid hormones (fT4 0,62 ng/dl with normal values between 0.70 and 1.80, fT3 1,32 pg/ml with normal values between 1.7 and 5.2) and normal levels of thyroid-stimulating hormone, without any clinical sign of hypothyroidism. Because of the aggravation of the renal disease, the patient was transferred to another hospital where he continued human albumin replacement therapy and cyclophosphamide and than he began haemodiafiltration. During this period he was subjected to renal biopsy which demonstrated the presence of a focal and segmental glomerulonephritis with mesangial matrix proliferation and an increase of Immunoglobulines M. Currently the patient is undergoing peritoneal dialysis waiting for kidney transplantation.

## Conclusions

The significance of this study is due to the early onset of autoimmune thyroiditis in association with nephrotic syndrome in a child under one year of age. The association found in our patient seems to be random because nephrotic syndrome does not have an autoimmune pathogenesis and there is no genetic or antigenic relation between autoimmune thyroiditis and nephrotic syndrome [8-10]. Finally, the case described suggests that serum thyrotropin and thyroxin levels should be determined in all cases of renal failure, even if the patient does not present the clinical signs of hypothyroidism.

## Consent

Written informed consent was obtained from the patient’s parents for publication of this case report and any accompanying images.

## Competing interests

The authors have indicated they have no relationships relevant to this article to disclose.

## Authors’ contributions

FU carried out the endocrinological and nephrological studies, drafted the manuscript and got the image. AA carried out the molecular genetic and pathogenetic studies. GA participated in the sequence alignment. RP provided the literature. LC coordinated the work. All authors read and approved the final manuscript.
